# Birth Mode Does Not Determine the Presence of Shared Bacterial Strains between the Maternal Vaginal Microbiome and the Infant Stool Microbiome

**DOI:** 10.1128/spectrum.00614-23

**Published:** 2023-06-20

**Authors:** Scott J. Dos Santos, Ishika Shukla, Janet E. Hill, Deborah M. Money, The Maternal Microbiome Legacy Project Team

**Affiliations:** a Department of Veterinary Microbiology, Western College of Veterinary Medicine, University of Saskatchewan, Saskatoon, Saskatchewan, Canada; b Department of Obstetrics and Gynaecology, Faculty of Medicine, University of British Columbia, Vancouver, British Columbia, Canada; c Department of Microbiology and Immunology, Faculty of Science, University of British Columbia, Vancouver, British Columbia, Canada; d Women’s Health Research Institute, B.C. Women’s Hospital, Vancouver, British Columbia, Canada; University of Nevada Reno

**Keywords:** birth mode, stool microbiome, vaginal microbiome

## Abstract

Dysbiosis of the neonatal gut microbiome during early life has been suggested as the missing link that may explain higher rates of certain diseases in caesarean section-delivered infants. Many studies report delivery mode-related dysbiosis in infants due to a lack of maternal vaginal microbiome exposure, prompting interventions to correct the neonatal gut microbiome by transferring these missing microbes after caesarean delivery. The maternal vaginal microbiome is among the first microbial exposures that many infants experience, yet little is known about the extent of direct transmission of maternal vaginal microbes. As part of the Maternal Microbiome Legacy Project, we aimed to determine if maternal vaginal bacteria are vertically transmitted to infants. We employed *cpn*60 microbiome profiling, culture-based screening, molecular strain typing, and whole-genome sequencing to determine whether identical maternal vaginal strains were present in infant stool microbiomes. We identified identical *cpn*60 sequence variants in both halves of maternal-infant dyads in 204 of 585 Canadian women and their newborn infants (38.9%). The same species of *Bifidobacterium* and *Enterococcus* were cultured from maternal and corresponding infant samples in 33 and 13 of these mother-infant dyads, respectively. Pulsed-field gel electrophoresis and whole-genome sequencing determined that near-identical strains were detected in these dyads irrespective of delivery mode, indicating an alternative source in cases of caesarean delivery. Overall, we demonstrated that vertical transmission of maternal vaginal microbiota is likely limited and that transmission from other maternal body sites, such as the gut and breast milk, may compensate for the lack of maternal vaginal microbiome exposure during caesarean delivery.

**IMPORTANCE** The importance of the gut microbiome in human health and disease is widely recognized, and there has been a growing appreciation that alterations in gut microbiome composition during a “critical window” of development may impact health in later life. Attempts to correct gut microbiome dysbiosis related to birth mode are underpinned by the assumption that the lack of exposure to maternal vaginal microbes during caesarean delivery is responsible for dysbiosis. Here, we demonstrate that there is limited transmission of the maternal vaginal microbiome to the neonatal gut, even in cases of vaginal delivery. Furthermore, the presence of identical strains shared between mothers and infants in early life, even in cases of caesarean delivery, highlights compensatory microbial exposures and sources for the neonatal stool microbiome other than the maternal vagina.

## INTRODUCTION

Over the past 2 decades, appreciation of the role played by the gut microbiome in health and disease has grown, and there are a myriad of studies showing an association between dysbiosis in the gut and diseases such as asthma, type I diabetes, atopy, and various chronic and autoimmune disorders (1–5). Of note, it has been suggested that gut dysbiosis in early life may be associated with increased predisposition to such diseases (6, 7). This notion is supported by evidence that gut microbiomes are involved in the generation of immune tolerance (8, 9) and general development of innate (10, 11) and adaptive immunity (12–14).

It has been speculated that early life gut microbiome dysbiosis might mediate the link between caesarean section (CS) delivery and the higher rates of several aforementioned diseases reported among infants delivered by CS (15, 16). Small-scale reports (17) of delivery mode-related dysbiosis in gut microbiomes of CS-delivered infants have since been corroborated by larger studies involving hundreds to thousands of infants (18, 19), leading some researchers to suggest interventions to “correct” this dysbiosis. One such practice is vaginal seeding (20), in which the mouth, nose, and skin of CS-delivered newborn infants is wiped with cotton gauze containing vaginal fluid collected from the mother with the aim of transferring maternal vaginal microbes. Although currently unproven and not recommended by obstetric and gynecological societies (21), this practice is predicated on the idea that exposure to the maternal vaginal microbiome is important for the establishment and “normal” development of the infant gut microbiome. At present, there is little evidence demonstrating a proven benefit of vaginal seeding (20–22), though clinical trials are under way.

The effect exerted by a mother’s vaginal microbiome on infant gut microbiome development is understudied, although our recent study of over 600 women and their infants (23) found that the overall composition of the maternal vaginal microbiome prior to delivery did not affect the trajectory of infant gut microbiome development, even among vaginally delivered infants. However, there remains a possibility that a subset of the maternal vaginal microbiota could be transferred vertically to the neonatal gut and seed the nascent microbial community or affect subsequent microbial succession. Current evidence suggests that this is possible (24, 25), though studies are either small in scale, do not directly study maternal vaginal organisms (i.e., inference of maternal origin), or do not demonstrate that the same evidence for vertical transfer is absent in CS-delivered infants. In contrast, transmission of specific strains from the maternal gut and breast milk has been repeatedly demonstrated (25–28).

Here, we aimed to investigate potential transmission of bacteria from the maternal vaginal microbiome to the infant gut microbiome. Specifically, we sought to determine if identical strains of bacteria are present in the maternal vagina and infant stool and if the presence of shared strains differs with birth mode (i.e., exposure to the vaginal microbiome). A notable difference from previous investigations is that our study population included vaginal deliveries, elective CS deliveries, and emergency CS deliveries, the latter population potentially being exposed to the maternal vaginal microbiome following membrane rupture and attempted labor. We performed analysis of *cpn*60 amplicon sequence data to examine overall relationships between vaginal and stool microbiome compositions and isolated *Bifidobacterium* and *Enterococcus* strains from mother-infant dyads for strain-level analysis. The results were interpreted in the context of considering the justification for vaginal seeding of CS-delivered infants to “compensate” for missing exposure to their mothers’ vaginal microbiota.

## RESULTS

### Potential transfer of *cpn*60 ASVs within mother-infant pairs.

**FIG 1:**
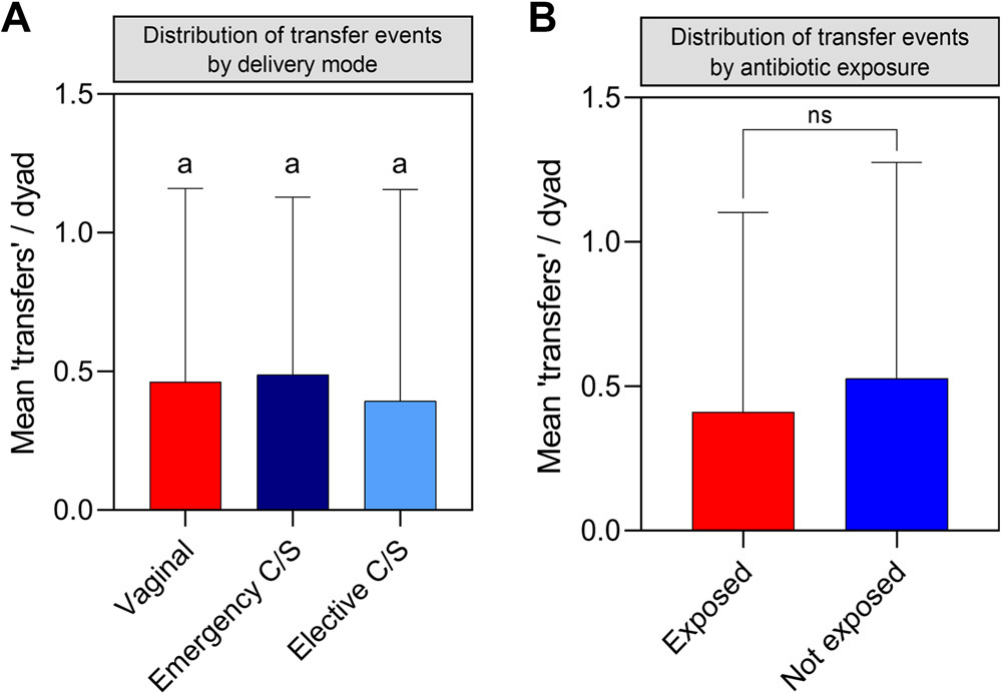
Delivery mode and intrapartum antibiotic exposure do not affect the number of “transfer” events per dyad. A potential transfer event was defined as detection of the same ASV in a maternal vaginal microbiome and at least one stool microbiome from the corresponding infant samples. The mean number of potential transfers per dyad ± standard deviation (SD) was calculated for mother-infant dyads grouped by mode of delivery (A) and maternal intrapartum antibiotic administration (B). Identical letters above error bars indicate no significant difference between groups; ns, not significant (Kruskal-Wallis test with Dunn's multiple-comparison correction). (A) *P* > 0.328; (B) *P* = 0.706.

Our data set of 585 maternal vaginal microbiomes and 1,027 infant stool microbiomes (568 10-day-old infants and 459 3-month-old infants) contained 7,717 amplicon sequence variants (ASVs), of which 3,794 were detected in maternal vaginal microbiomes (see File S1 at https://doi.org/10.6084/m9.figshare.22063148.v1) and could thus be tracked between mothers and infants. Among these 585 mother-infant dyads with microbiome profiling data for the maternal vaginal microbiome and at least one infant stool microbiome, we identified a total of 260 instances where the same ASV was detected in the maternal vaginal microbiome and at least one corresponding infant stool microbiome at a relative abundance of ≥0.1%, indicative of a potential “transfer” event (see File S3 at https://doi.org/10.6084/m9.figshare.22063148.v1). There were 147 such instances where the same ASV was present in the mother and her 10-day-old infant, 72 where ASVs were present in the mother and her 3-month-old infant, and 41 instances where the ASV was present in all three microbiome profiles (i.e., sustained presence in the infant during early life). Across the cohort, “transfer” of at least one ASV was observed in 204 of 585 mother-infant dyads (34.9%; range, 1 to 5 ASVs; median, 1 ASV). The distribution of these potential transfer events did not differ based on delivery mode (Fig. 1A), with mean “transfer” events per dyad of 0.46 for vaginally delivered infants, 0.49 for infants delivered by emergency CS, and 0.40 for infants delivered by elective CS (*P* > 0.328, Kruskal-Wallis test with Dunn’s multiple-comparison correction). Maternal intrapartum antibiotic exposure was found to be a confounder of delivery mode-related dysbiosis among infant stool microbiomes in the Maternal Microbiome Legacy Project study; however, no significant differences in the mean number of “transfer” events was noted based on intrapartum antibiotic exposure in the present study (Fig. 1B).

Of the ASVs potentially transferred from mother to infant, the 10 most frequently “transferred” included typical vaginal organisms such as *Lactobacillus* spp. and those associated with the vaginal environment such as *Bifidobacterium* spp. and *Actinomyces* sp. These 10 ASVs accounted for 173 of 260 (65.4%) “transfers” (Table 1). Identical ASVs of *Lactobacillus* spp. (which comprise the vast majority of organisms of the vaginal microbiome in most women of reproductive age [29, 30]) were largely identified in maternal vaginal microbiomes and 10-day-old infant stool microbiomes, reflecting their transient presence in the neonatal gut in the earliest days to weeks of life (31). Similarly, the seven *Lactobacillus* ASVs potentially transferred from mother to infant were detected at a relative abundance of >0.1% a total of 984 times among the 585 maternal vaginal microbiomes (see Table S1 in the supplemental material), with a total of 89 “transfers” (9.0% transfer rate). Lactobacillus crispatus accounted for 61 of 89 (68.5%) of these potential transfers.

**TABLE 1:** Taxonomic assignments of the 10 most frequently “transferred” ASVs and percent sequence similarity of each ASV to their corresponding closest match in cpnDB^*a*^

ASV ID	Taxonomic assignment	% Identity	No. of maternal samples > threshold	No. of “transfers”	No. of infant samples > threshold at indicated time point(s)		
					Both	10 days	3 mo
1	Lactobacillus crispatus	100	380	61	0	57	4
**2**	**Bifidobacterium breve**	**98**	**43**	**28**	**11**	**7**	**10**
3	Actinomyces neuii subsp. *anitratus*	100	232	23	1	16	6
**4**	**Bifidobacterium longum subsp. *longum***	**100**	**25**	**15**	**5**	**5**	**5**
5	Lactobacillus gasseri	100	146	12	0	7	5
6	Shigella sonnei	98.7	23	12	6	3	3
7	Escherichia coli	100	15	8	2	2	4
8	Lactobacillus jensenii	100	203	7	0	7	0
**9**	**Bifidobacterium dentium**	**98.7**	**16**	**4**	**0**	**3**	**1**
10	Escherichia coli	99.3	9	3	0	3	0

a The number of maternal samples above the “transfer” threshold and the number of “transfer” events across the data set are indicated in addition to the detection of the same ASVs in infant stool microbiomes of the corresponding infants. ASV identification numbers (IDs) are not consistent across tables. Bifidobacterial ASVs are indicated in bold.

Bifidobacteria are known inhabitants of both the vagina and the gastrointestinal tract, and these populations are indistinguishable at the genomic level (32). Similarly, enterococci are also known commensals of the vaginal environment, although they are present at very low abundances (33, 34). Therefore, we also assessed potential transfer of these genera specifically. The 17 bifidobacterial ASVs undergoing potential transfer (Table 2) were detected a total of 137 times across all maternal vaginal microbiomes and accounted for a total of 69 “transfers” (50.4% transfer rate). Of these 69 “transfers,” 28 involved ASVs aligning to Bifidobacterium breve (40.6%) and 15 were attributed to an ASV aligning to Bifidobacterium longum subsp. *longum* (21.7%). “Transfer” of these ASVs was routinely observed in both 10-day-old and 3-month-old infant stool microbiomes, with persistence of these ASVs within infant stool microbiomes over time in 20 of 69 cases (29.0%).

**TABLE 2:** Taxonomic assignments for ASVs aligning to *Bifidobacterium* spp. and their percent sequence similarity to the closest match in cpnDB^*a*^

ASV ID	Taxonomic assignment	% Identity	No. of maternal samples > threshold	No. of “transfers”	No. of infant samples > threshold at indicated time point(s)		
					Both	10 days	3 mo
1	Bifidobacterium adolescentis	100	1	1	0	1	0
2	Bifidobacterium adolescentis	98.7	1	1	0	0	1
3	Bifidobacterium bifidum	98.7	4	2	0	0	2
**4**	**Bifidobacterium breve**	**98**	**43**	**28**	**11**	**7**	**10**
5	Bifidobacterium breve	98.7	6	3	2	1	0
6	Bifidobacterium breve	97.3	3	3	0	2	1
7	Bifidobacterium breve	97.3	4	2	0	0	2
8	Bifidobacterium breve	98	10	1	0	1	0
9	Bifidobacterium breve	98	1	1	1	0	0
10	Bifidobacterium breve	98	1	1	0	1	0
11	Bifidobacterium breve	97.3	2	1	0	0	1
**12**	**Bifidobacterium dentium**	**98.7**	**16**	**4**	**0**	**3**	**1**
13	Bifidobacterium longum subsp. *infantis*	100	2	1	1	0	0
**14**	**Bifidobacterium longum subsp. *longum***	**100**	**25**	**15**	**5**	**5**	**5**
15	Bifidobacterium longum subsp. *longum*	99.3	12	3	0	3	0
16	Bifidobacterium longum subsp. *longum*	99.3	3	1	0	0	1
17	Bifidobacterium pseudocatenulatum	99.3	3	1	0	1	0

a Number of maternal samples above the 0.1% “transfer” threshold and number of “transfers” across the data set are indicated for each ASV, along with their detection in infant stool. ASVs from Table 1 are indicated in bold (top 10 most frequently “transferred”).

For enterococcal ASVs, “transfer” was observed much less often, owing to the extremely low abundance of this genus in the vaginal microbiome. Only three potentially transferred ASVs were identified (see Table S1 in the supplemental material), detected a total of 47 times across 585 maternal vaginal microbiomes. Only seven cases of “transfer” could be observed, three for Enterococcus casseliflavus and four for Enterococcus faecalis.

### Molecular typing highlights close similarity of strains within dyads.

To corroborate ASV-based evidence for “transfer” of maternal vaginal microbiota, we undertook selective culture of maternal vaginal swab fluid and infant stool samples to isolate viable bifidobacteria and enterococci and compare the relatedness of isolates within and between mother-infant dyads. These genera are constituents of both vaginal and stool microbiomes and can be readily isolated on selective culture media. Seventy-nine dyads met the screening criteria for cultivation on Bifidus selective medium (BSM) or m-*Enterococcus* (m-Ent) agar, and a total of 499 isolates were recovered. Of these isolates, 481 (96.4%) were identified by PCR amplification and Sanger sequencing of the 16S rRNA gene. Bifidobacterial species typical of the vagina and gut environments comprised half of the culture collection (243 of 481; 50.5%), with a preponderance of B. breve and B. longum subsp. *longum* (Table 3).

**TABLE 3:** Isolates cultured from maternal vaginal swab fluid and infant stool samples on BSM or m-Ent agar (*n* = 481 of 499, 96.4%) and identified by Sanger sequencing of the 16S rRNA gene and taxonomic assignment using the RDP SeqMatch tool^*a*^

Species identity	No. of isolates	No. of dyads
Bifidobacterium breve	154	25
Bifidobacterium longum subsp. *longum*	46	4
Bifidobacterium longum subsp. *infantis*	5	1
Bifidobacterium dentium	10	1
Bifidobacterium bifidum	11	2
Bifidobacterium pseudocatenulatum	11	0
*Bifidobacterium* sp.	6	0
Lactobacillus rhamnosus	21	1
Lactobacillus casei */paracasei*	12	1
Lactobacillus plantarum	1	0
Enterococcus faecalis	135	13
Enterococcus gallinarum	8	0
Enterococcus durans	1	0
Enterococcus faecium	3	0
Enterococcus avium	3	0
*Winkia* (*Actinomyces*) *neuii*	4	0
Streptococcus agalactiae	15	0
Staphylococcus epidermidis	23	1
Propionibacterium lymphophilum	5	0
Other species	7	0

a The numbers of isolates and instances for which the same species was isolated from a mother and corresponding infant are indicated.

*Lactobacillus* spp., and to a greater degree enterococci, were also recovered in number. Overall, we identified 33 mother-infant pairs with a shared total of 143 *Bifidobacterium* isolates cultivated from both halves of the dyad, and 13 pairs where 64 Enterococcus faecalis isolates were recovered from maternal and corresponding infant samples. There was high concordance between microbiome profiling and culture screening data for *Bifidobacterium*: 28 of 33 dyads (84.9%) from which viable bifidobacteria were recovered from mothers and infants also exhibited identical ASVs of the same species in the maternal vaginal and corresponding infant stool microbiomes. For E. faecalis isolates, agreement between culture and ASV transfer data was limited to 2 of 13 dyads (15.4%), owing to the low abundance of this species in vaginal microbiomes and high sensitivity of bacterial culture (see Table S1 in the supplemental material; see also File S1 at https://doi.org/10.6084/m9.figshare.22063148.v1).

Isolates of *Bifidobacterium* spp. and Enterococcus faecalis cultured from both maternal and infant samples were subjected to strain typing by pulsed-field gel electrophoresis (PFGE) (see Fig. S1 in the supplemental material for representative gels). Visual analysis of PFGE banding patterns revealed that identical banding patterns were shared between isolates from the same mother-infant dyad regardless of delivery mode (Table 4) for both *Bifidobacterium* spp. and E. faecalis isolates. Furthermore, there was no significant difference in the distribution of the number of dyads whose isolates exhibited identical or nonidentical banding patterns among delivery modes for either species (Yates corrected chi-square test, *P = *0.570 and 0.359, respectively) or intrapartum antibiotic exposure (*P = *0.601 and 0.305, respectively). However, bifidobacterial isolates recovered from 10-day-old infants were more likely to have a banding pattern identical to a corresponding maternal vaginal isolate than those from 3-month-old infants (*P* < 0.01). Agreement of ASV “transfer” data were less consistent with PFGE banding patterns, with only 21 of 33 dyads (63.6%) showing agreement between the presence of shared bifidobacterial ASVs between maternal and infant microbiomes and identical banding patterns shared between isolates of the corresponding species from the same dyad. For enterococci, agreement between ASV data and PFGE banding patterns was observed for only 2 of 13 dyads (15.4%).

**TABLE 4:** Summary of PFGE strain typing by mode of delivery^*a*^

Species identified	Parameter	No. of dyads or isolates			% Identity	*P*
		Identical	Nonidentical	Total		
*Bifidobacterium* spp. (*n* = 33)	Delivery mode (no. of dyads)					
	Vaginal	10	5	15	66.7	0.570
	CS elective	7	5	12	58.3	
	CS emergency	5	1	6	83.3	
	IP antibiotics (no. of dyads)					
	Unexposed	8	3	11	72.7	0.601
	Exposed	14	8	22	63.6	
	Time point (no. of isolates)					
	10 days	34	9	43	79.1	<0.01
	3 mo	24	21	45	53.3	
						
Enterococcus faecalis (*n* = 13)	Delivery mode (no. of dyads)					
	Vaginal	3	0	3	100.0	0.359
	CS elective	2	2	4	50.0	
	CS emergency	4	2	6	66.7	
	IP antibiotics (no. of dyads)					
	Unexposed	2	0	2	100	0.305
	Exposed	7	4	11	63.6	
	Time point (no. of isolates)					
	10 days	16	8	24	66.7	0.710
	3 mo	11	7	18	61.1	

a Strain typing was performed on all isolates of *Bifidobacterium* spp. and Enterococcus faecalis where the same species was identified in both halves of maternal-infant dyads. Interpretation of PFGE banding patterns is summarized in terms of identical or nonidentical patterns between isolates from the same dyad, grouped by delivery mode, intrapartum antibiotic exposure (IP antibiotics), and infant stool sample collection time point. Differences in distribution of identical and nonidentical banding patterns were assessed by Yates-corrected chi-square test.

### The presence of identical maternal and infant B. breve strains is not limited by delivery mode.

Having demonstrated that identical strains appeared to be present in both vaginally and CS-delivered infants, we sought to confirm if the observations from PFGE could be corroborated by genome sequencing and higher-resolution strain-level analysis. Therefore, we selected 35 isolates of *Bifidobacterium* spp. for whole-genome sequencing (30 B. breve, 3 B. longum, and 2 B. bifidum isolates), representing 12 dyads, all three delivery modes, and instances of both identical (*n *= 10) and nonidentical (*n *= 2) banding patterns shared between infant isolates and at least one corresponding maternal isolate. No infants undergoing vaginal delivery involved in this analysis were exposed to intrapartum antibiotics, meaning that the comparisons between delivery mode and antibiotic exposure groups are equivalent. We assembled 35 draft-level genomes with an average coverage of 69× and an average *N*_50_ of 595,126 bp (see Table S2 in the supplemental material). Following removal of unclassified and nonbifidobacterial contigs (see Fig. S2 in the supplemental material), an average of 35 ± 18 bifidobacterial contigs per assembly were available for analysis. As expected for bifidobacteria (26, 32), all genomes exhibited high G+C content (58.6 to 62.64%) and assembly lengths were consistent with complete reference genomes for all three species (2.19 to 2.38 Mb).

**FIG 2:**
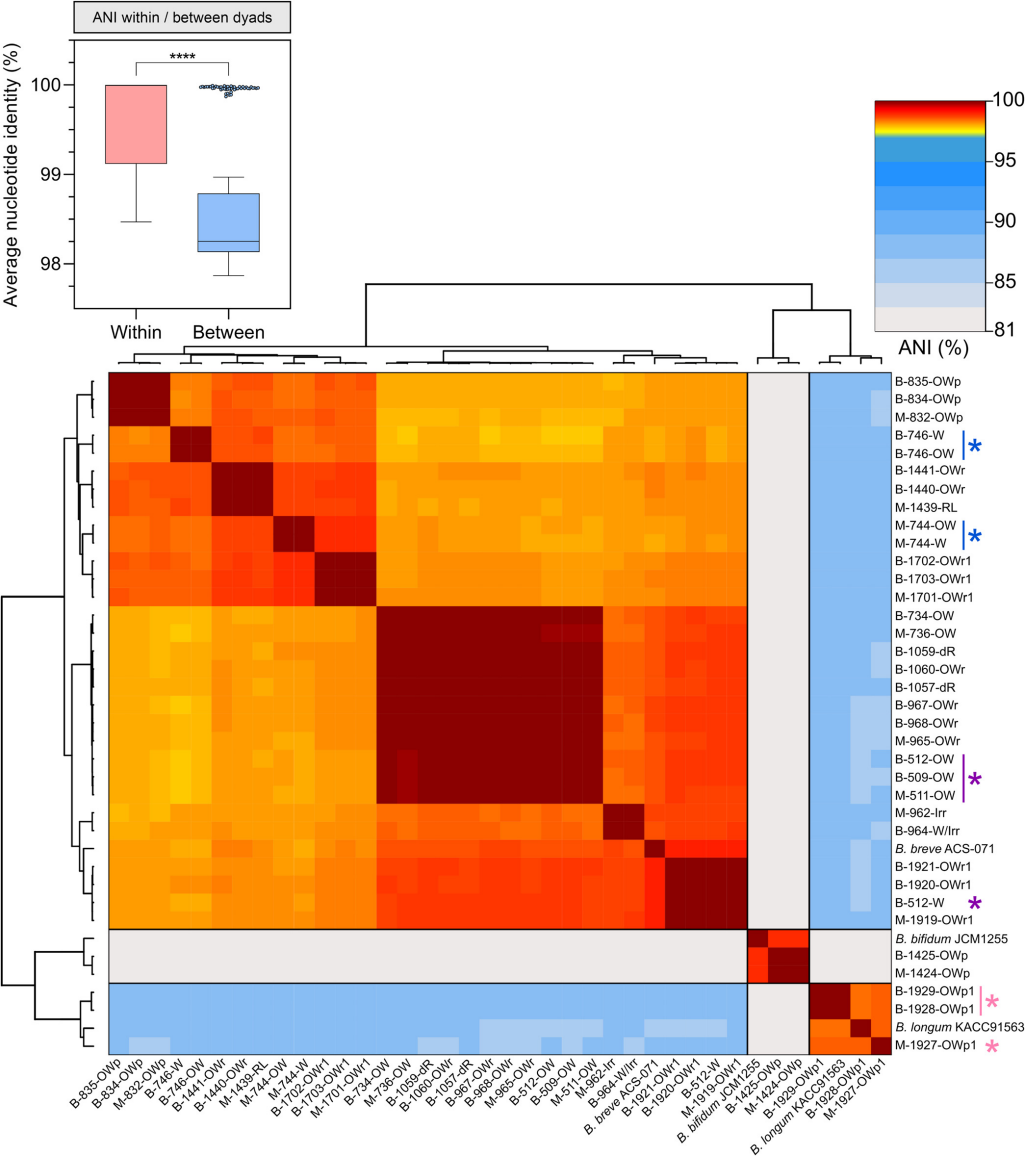
Genomes of bifidobacterial isolates are more similar within mother-infant dyads than between dyads based on ANI. Pairwise ANI was calculated for all 35 draft-level assemblies, plus complete reference genomes for each of the three species (main plot). Colored asterisks indicate genomes where ANI values for maternal (M) and baby (B) isolates suggest no relatedness. Distribution of ANI values when isolates within and between maternal-infant dyads are compared is also shown (inset plot; ****, *P* < 0.0001, Mann-Whitney U test). Bars indicate median, and whiskers indicate 1.5× interquartile range.

Pairwise comparison of average nucleotide identities (ANIs) across whole genomes delineated the three species of *Bifidobacterium*, with approximately 81% ANI between B. bifidum and both B. breve and B. longum subsp. *longum* and approximately 87% ANI for comparisons between B. breve and B. longum subsp. *longum* (Fig. 2). Even within clusters of highly similar B. breve isolates, comparison of pairwise ANIs revealed almost identical ANIs for isolates from the same mother-infant dyad (>99.99% ANI) (see File S4 at https://doi.org/10.6084/m9.figshare.22063148.v1), and the median within-dyad ANI was significantly higher than the between-dyad ANI (Fig. 2, 99.998% versus 98.251%; *P* < 0.0001, Mann-Whitney U test). ANI values perfectly reflected PFGE data such that isolates with identical banding patterns exhibited ANI values of >99.9%, while those with nonidentical banding patterns showed lower ANIs (~98 to 99%).

**FIG 3:**
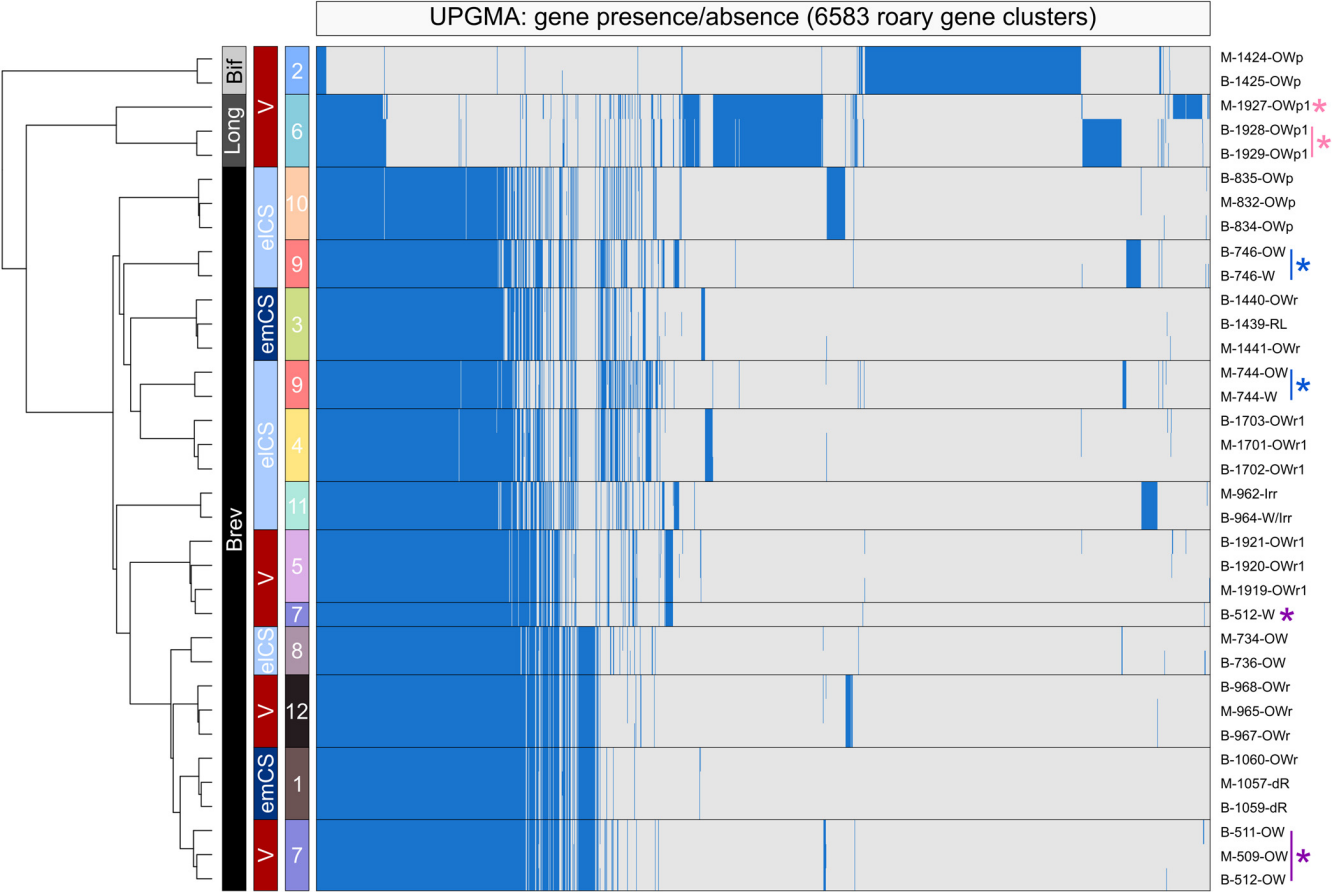
Pangenome analysis reveals that high genetic relatedness between maternal and infant isolates is not restricted to vaginally delivered infants. Pangenome analysis was conducted by Roary using Prokka-annotated genomes as input. UPGMA clustering was performed on the resultant gene cluster presence/absence matrix. Colored asterisks indicate genomes where ANI values and gene presence/absence for maternal (M) and baby (B) suggest no relatedness. Side color bars indicate the following (left to right): species, delivery mode, and dyad number. Brev, B. breve; Long, B. longum subsp. longum; Bif, B. bifidum; V, vaginal; elCS, elective CS; emCS, emergency CS.

We next conducted annotation of these 35 genomes: the mean number of genes identified for isolates of B. breve (*n* = 1,938), B. longum subsp. *longum* (*n* = 1,917), and B. bifidum (*n *= 1,742) agreed well with reference genomes and previously published data (26, 32), and the percentage of genes encoding proteins annotated as “hypothetical” ranged from 17.9% to 25.2%. Pangenome analysis demonstrated the presence of extremely similar gene cluster profiles within dyads, consistent with both PFGE and ANI data (Fig. 3). Isolates from the three different species clustered separately as expected; however, isolates from mother-infant pairs that exhibited identical banding patterns and high ANI values also clustered into their respective dyads regardless of delivery mode. Likewise, notable differences in gene content were seen for dyads showing different banding patterns or lower ANI values (e.g., 1927-OWp1 versus 1928-OWp1 and 1929-OWp1).

**FIG 4:**
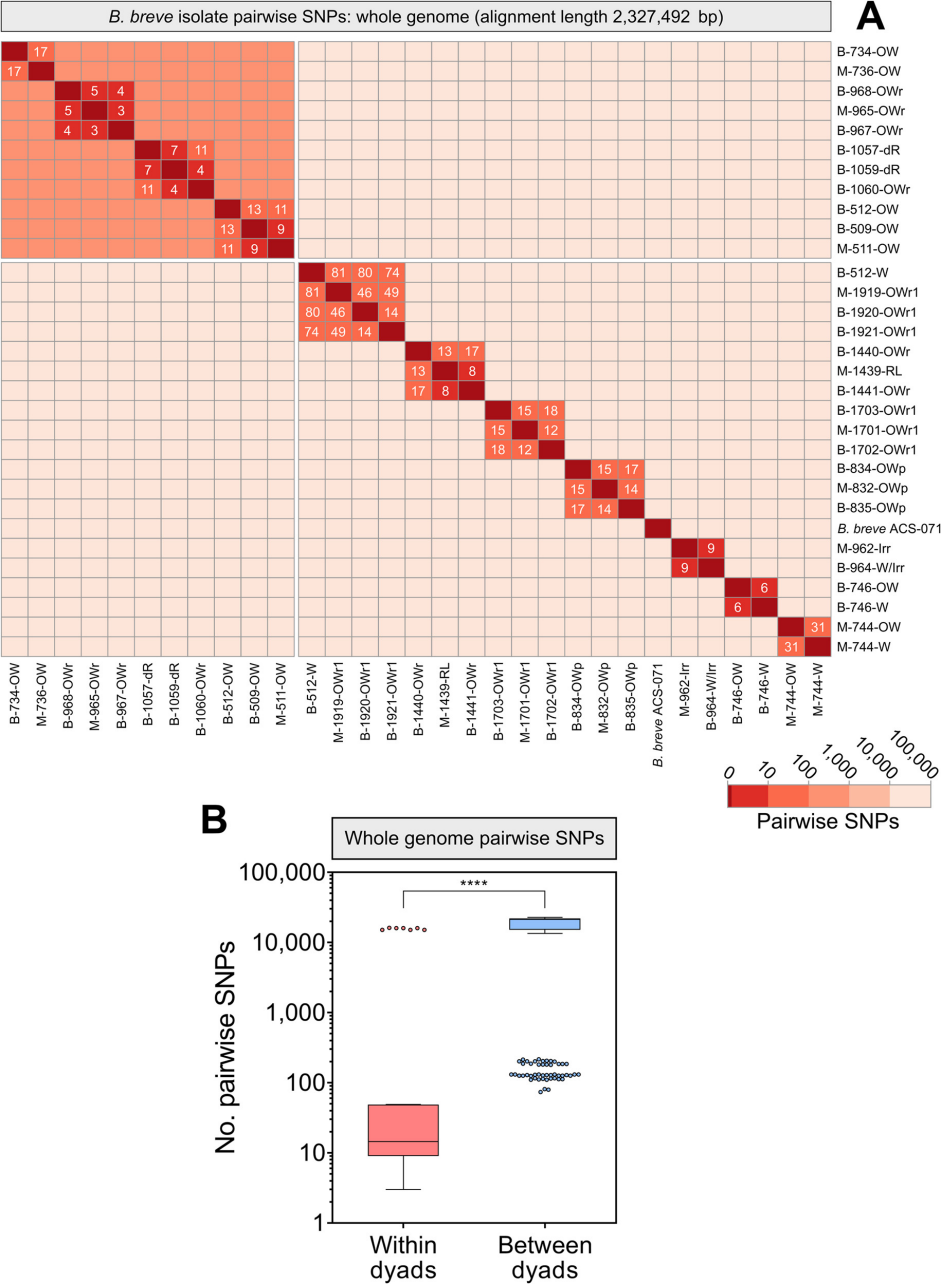
SNP analysis demonstrates near-identical B. breve isolates within mother-infant dyads. Multiple whole-genome alignment and SNP analysis were performed for all B. breve draft genomes using Snippy. (A) A pairwise comparison matrix of SNPs between all genomes was constructed: self-comparisons are indicated by dark red boxes (0 SNPs). (B) Number of SNPs for all comparisons within (red) and between (blue) mother-infant pairs. Bar indicates median number of SNPs, and whiskers indicate 1.5× interquartile range (****, *P* < 0.0001, Mann-Whitney U test).

**FIG 5:**
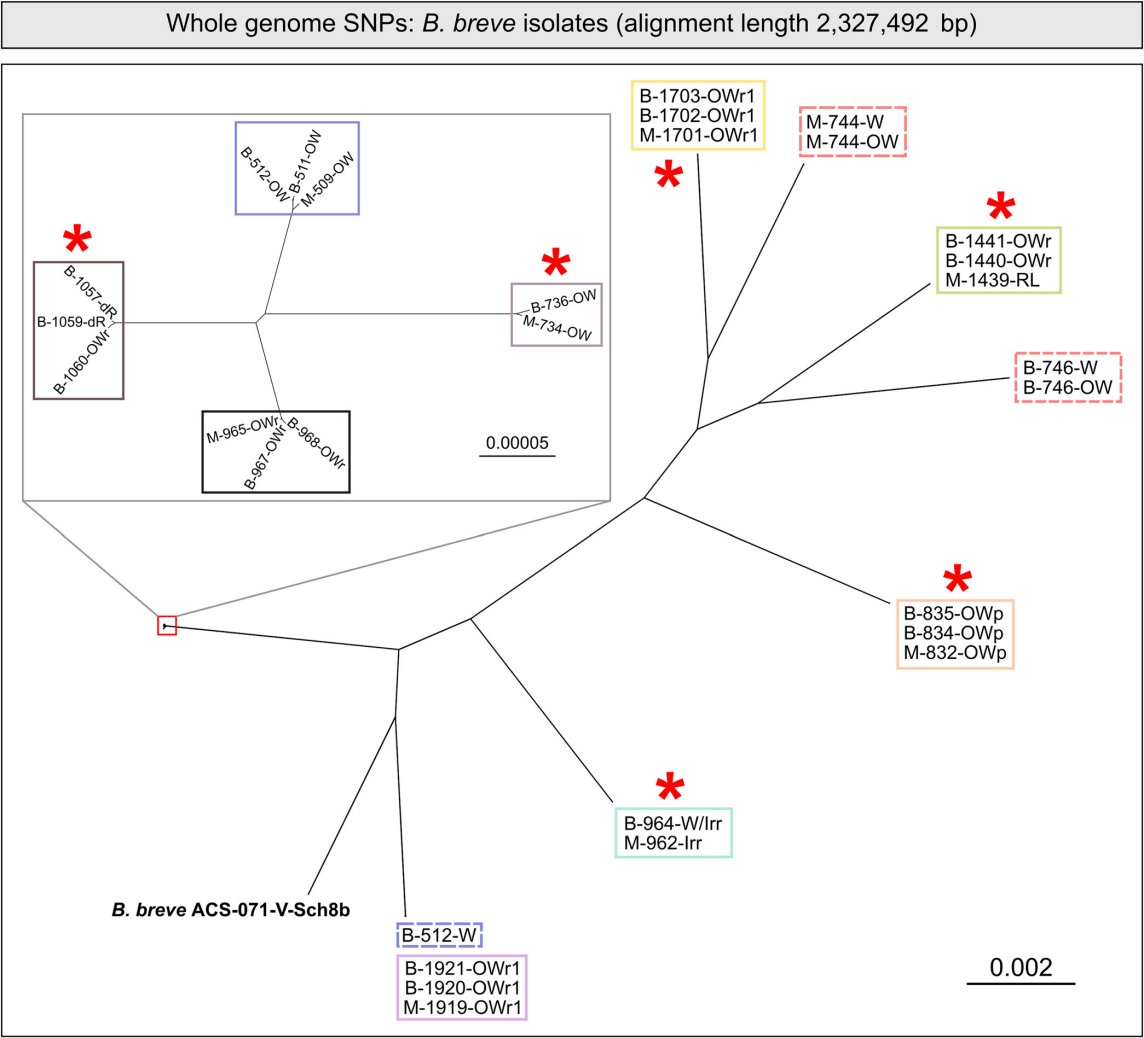
Identical isolates of B. breve are found in maternal-infant dyads regardless of delivery mode. A phylogenetic tree was constructed from the whole-genome alignment, with near-identical isolates from the same dyads highlighted by colored boxes. Broken lines indicate unrelated isolates from the same dyad. Asterisks indicate dyads harboring near-identical dyads despite CS delivery. Scale bar indicates nucleotide substitutions per site.

Finally, we investigated differences in single-nucleotide polymorphisms (SNPs) between the 30 B. breve genomes. Studies assessing pathogen transmission in outbreak settings using diagnostic sequencing typically involve thresholds dictating the maximum number of SNPs separating any two given genomes to define transmission events. While thresholds differ by species, the upper bound of whole-genome SNP thresholds is approximately 15 to 25 SNPs (35–37). Overall, pairwise comparison of SNPs between all B. breve genomes recapitulated prior findings of within-dyad relatedness between maternal and infant isolates (Fig. 4A). Within dyads showing identical banding patterns and highly similar genomes based on ANI and pangenome analysis, we routinely observed fewer than 20 SNPs separating maternal and infant isolates. Conversely, isolates showing different banding patterns and less-similar genomes routinely exhibited upwards of 20,000 SNPs (see File S4 at https://doi.org/10.6084/m9.figshare.22063148.v1). Overall, there were significantly fewer SNPs between isolates within the same mother-infant dyad than between dyads (median of 15 SNPs within dyad versus 21,362 between dyads) (Fig. 4B, *P* < 0.0001, Mann-Whitney U test). This same pattern was observed even in a subcluster of closely related B. breve isolates which showed pairwise ANI values of >99.9% for all comparisons and extremely similar gene cluster profiles (median of 8 SNPs within versus 152 SNPs between) (see Fig. S3 in the supplemental material). Similar to the pangenome analysis, near-identical SNP profiles were seen among isolates within dyads where infants were delivered vaginally as well as by CS (Fig. 5).

## DISCUSSION

Using a combination of culture-independent and culture-based approaches, we have shown that strains of *Bifidobacterium* spp. and E. faecalis detected in maternal vaginal microbiomes are detected in the stool of corresponding infants, potentially indicative of transmission. This phenomenon is not, however, restricted to infants exposed to the vaginal microbiota during delivery, which indicates other maternal sources for seeding the infant gut microbiome.

One likely explanation for the presence of near-identical bifidobacterial strains in the maternal vagina and infant gut in cases of CS delivery is that these isolates originated not from the maternal vagina but from maternal breast milk. *Bifidobacterium* spp. can be readily isolated from human breast milk (25, 26, 28, 38) and are commonly identified as constituents of the breast milk microbiome (39–41), despite the generally low total microbial biomass of this sample type. In this study, all infants from whom bifidobacteria were isolated, strain typed, and sequenced were either partially or exclusively breastfed at both infant sampling time points. Furthermore, many prior studies have identified breast milk as a vector for bifidobacterial transmission to the neonatal infant gut. Duranti et al. (26) employed a combination of amplicon sequencing, isolate-targeted whole-genome sequencing, and shotgun metagenomics to demonstrate the presence of shared bifidobacterial operational taxonomic units between breast milk samples and infant feces, as well as a high degree of concordance between bifidobacterial (meta)genomes originating from both environments within mother-infant pairs. Similar results have been reported by others: Yan and colleagues (42) recently reported concordant *Bifidobacterium* profiles within mother-infant dyads based on shared *cpn*60 ASVs in breast milk and infant stool microbiomes. Likewise, studies using techniques such as PFGE or amplified fragment length polymorphism analysis also report identical strains of bifidobacteria shared within, but not between, dyads (28, 38). It has been shown that bifidobacteria isolated from the gut and vagina, as well as those isolated from the gut and breast milk, are genomically indistinguishable (32); therefore, it is reasonable to speculate that detection of *Bifidobacterium* strains in CS-delivered infants that are practically identical to those from the maternal vagina in this study are due to transfer from maternal breast milk.

In cases of vaginal delivery or emergency CS with prolonged labor, we cannot rule out the possibility of transmission from the maternal vagina in our study. Others have described similar results, suggesting a maternal vaginal origin for bifidobacterial isolates cultured from the infant gut in early life. Toda and coworkers (24) cultivated *Bifidobacterium* spp. from neonatal oral fluid (containing maternal vaginal secretions) collected immediately after delivery. They noted extremely high pairwise ANI values for oral fluid bifidobacteria and those cultured from infant feces at approximately 1 month postpartum, although no maternal bifidobacteria were explicitly studied. Ferretti et al. (25) investigated vertical transmission of maternal microbiota through high-depth shotgun metagenomic profiling of multiple maternal body sites, including the vagina. They also demonstrated vertical transmission of multiple different vaginal species, including *Bifidobacterium* spp., to the infant gut. While consistent with our results, these studies specifically excluded CS-delivered infants. More recently, Mortensen and colleagues (43) demonstrated the presence of shared maternal vaginal ASV levels in infant stool microbiomes regardless of delivery mode. While ASVs were grouped at the order level, their analysis using weighted transfer ratios reflected our own conclusions that “transfer” appears to be independent of delivery mode.

The vaginal microbiome of women of reproductive age contains an abundance of *Lactobacillus* spp. (29, 44) and, to a far lesser extent, *Bifidobacterium* spp. (though it can be a dominant member of the vaginal microbiome in a subset of women exhibiting asymptomatic bacterial vaginosis [45]). This was reflected in our culture screen of the Maternal Microbiome Legacy Project study participants on media designed for selective isolation of bifidobacteria and lactobacilli, though we noted the absence or extremely low abundance of vaginal lactobacilli in 10-day-old and (especially) 3-month-old infant stool microbiomes among the ASV data set. Their presence in the infant gut following birth, however transient, may have implications for development of immune tolerance, as demonstrated recently (46).

The recovery of many Enterococcus faecalis isolates from vaginal swab fluid samples was somewhat unexpected. Extremely low counts of E. faecalis ASVs were observed in vaginal microbiome profiles, and very few instances of potential ASV transfer were detected. E. faecalis is, however, a known colonizer of the female genitourinary tract (47–49) and has been associated with both aerobic vaginitis (50) and commensal carriage (33, 34), though it is understudied in the latter context. As mentioned above, we also identified identical strains of E. faecalis in mothers and CS-delivered infants by PFGE, which cannot be explained by transmission from the maternal vaginal microbiome. Like bifidobacteria, enterococci have been detected in breast milk microbiomes (39) (albeit at very low abundances) and have also been isolated from breast milk itself (49), with one potential probiotic strain of E. faecium originating from breast milk (51). Accordingly, transmission via breastfeeding remains a feasible explanation for our results.

Our data suggest that the vaginal microbiome is unlikely to be a major contributor to the transmission of maternal microbiota to the neonatal gut. Colonization of the infant gut by transferred vaginal microbes is also inherently limited by the vastly different (and incompatible) physiological conditions of the neonatal gut in comparison to those of the maternal vagina, meaning that growth and persistence of most of these organisms are not likely. While exposure to the vaginal microbiome during birth may result in transmission, it is evident that there are alternative routes of transmission for organisms such as *Bifidobacterium* or *Enterococcus* which inhabit multiple maternal body sites. In addition to breast milk, there is strong evidence implicating the maternal fecal microbiome in vertical transmission of enteric strains to the infant during birth. Yassour et al. (27) carried out strain-level metagenomic profiling of maternal and infant fecal samples in a cohort of 44 infants and identified multiple transmission events, often involving *Bacteroides* spp. and *Bifidobacterium* spp. These findings are corroborated by other studies assessing vertical transfer of maternal gut microbes (26, 42, 52–54), including those concluding that the contribution of gut and breast milk organisms to vertical transfer vastly outweighs that of vaginal organisms (25). Notably, one such study (40) identified an instance of potential transfer within a CS-delivered infant whereby a specific B. breve strain was detected in the maternal feces, maternal breast milk, and infant feces, raising questions about gut translocation and enteromammary transit. As gut translocation of Pseudomonas aeruginosa has recently been demonstrated in a separate context (55), this remains an attractive, although as yet unproven, hypothesis.

Given that vaginal microbiomes appear to contribute minimally to vertical transmission and our previous demonstration that the maternal vaginal microbiome composition has no detectable effect on the longitudinal development of the infant gut microbiome within the first months of life (23), it is appropriate to reconsider the rationale for vaginal seeding practices aiming to “correct” the gut microbiome of infants delivered by CS. If vertical transfer from the vagina is not a significant influence on establishment of the infant gut microbiome, the efficacy of postpartum vaginal seeding is likely limited. In accordance with this, studies of vaginal seeding fail to routinely show a shift of CS-delivered infant gut microbiomes toward the composition of those delivered vaginally (20, 22). Accordingly, there is a need to focus on maternal sources other than the vagina when investigating establishment of the neonatal gut microbiome and subsequent health outcomes. This need is underscored by studies reporting compensatory effects of breastfeeding (56–58) and maternal fecal microbiome transplantation (59) on the development of neonatal gut microbiomes in CS-delivered infants.

The limitation of utilizing microbiome data, even at the ASV level, was clear in our study: agreement between ASV “transfer” data and PFGE banding patterns was only 64% when considering dyads in which bifidobacterial strain typing was performed. However, this was not unexpected, as a single gene is unlikely to provide sufficient information for strain-level discrimination or assessment of transmission. This serves to highlight the importance of whole-genome sequencing and shotgun metagenomics for these analyses. Similarly, our culture-based screen was biased by the strategy of picking a limited number of colonies from each plate for further analysis and may have prevented detection of further instances of identical species in both halves of maternal-infant dyads. However, exhaustive analysis of all colonies is impractical, laborious, and expensive.

Overall, we have demonstrated that the maternal vaginal microbiome is a potential source of certain bacteria seeding the infant gut microbiome in cases of vaginal delivery. However, the observation of identical strains in the maternal vagina and stool of C/S-delivered infants indicates that other sources, such as the maternal gut or breast milk, can provide the same organisms, compensating for the lack of exposure to the vaginal microbiota.

## MATERIALS AND METHODS

### Study population and data collection.

We recruited healthy, pregnant individuals delivering at term into the Maternal Microbiome Legacy Project across three hospitals in British Columbia, Canada, between March 2018 and March 2020 (BC Women’s Hospital + Health Centre, Vancouver; Surrey Memorial Hospital, Surrey; University Hospital of Northern BC, Prince George). Participants were recruited either during pregnancy or at admission for delivery. Informed consent for study participation was obtained, and participants were enrolled upon meeting the following criteria: >18 years of age, ≥37 weeks gestational age at delivery, no known major fetal anomalies, and singleton or twin gestation. Participants were excluded from the study if one or more of the following criteria were met: inability to provide informed consent, participation in drug or probiotic trials, triplet or higher-order gestation, placenta previa at delivery, and placental abruption. Ethics approval was granted by the University of British Columbia Children’s and Women’s Health Centre Research Ethics Board, harmonized with partner boards at Fraser Health and Northern Health (certificate no. H17-02253). Demographic and clinical data were collected by research staff via interview and medical chart review and stored in the Research Electronic Data Capture (REDCap) database securely hosted at BC Children’s Hospital Research Institute (60).

### Microbiome profiling and ASV tracking.

A subset of the microbiome profiling data from participants enrolled in the Maternal Microbiome Legacy Project (23) was reanalyzed for the current study; feature tables and corresponding metadata for this subset (read counts and proportional abundance) are included in File S1 at https://doi.org/10.6084/m9.figshare.22063148.v1. The current analysis includes 585 maternal vaginal microbiomes, 568 stool microbiomes from 10-day-old infants, and 459 stool microbiomes from 3-month-old infants. Sample collection has been described in detail elsewhere (23); briefly, maternal vaginal samples were collected via swabbing of the lateral vaginal wall and posterior fornix upon maternal admission to the hospital for delivery. Stool samples were self-collected from diapers by parents and refrigerated at 4°C until collection by the study team either the same day or following morning. All samples were transported to the processing laboratory in British Columbia, Canada, on ice and stored at −80°C until shipping to the microbiome profiling laboratory on dry ice. Raw sequence data are available from the NCBI Sequence Read Archive under BioProject no. PRJNA824125. Sample collection, DNA extraction, PCR amplification of the *cpn*60 universal barcode region, and sequencing on an Illumina MiSeq platform have been described previously (23, 61). Only forward (R1) reads were used for analysis, as read-pair overlap is not physically possible after index PCR due to the length of the amplicon (~740 bp). We have previously demonstrated, however, that 150 bp of the *cpn*60 barcode region is sufficient for accurate taxonomic identification to the species level (62).

PCR primers were removed from sequence reads using Cutadapt (63), and reads were trimmed for quality using Trimmomatic (64) (minimum Phred score, Q30 over a 4-bp sliding window; minimum length, 150 bp). Variant calling was performed by DADA2 (65) as part of the QIIME2 package (66) to generate 150-bp ASVs, and taxonomy was assigned by comparison to a nonredundant version of cpnDB (67) (cpnDB_nr_20220726) using wateredBLAST (44). ASVs with sequence similarity of <55% to a cpnDB entry were discarded. The ASV feature table was screened for contamination using the decontam (68) package in R running in “prevalence” mode with the “batch” option set, using the 191 negative controls implemented across the Maternal Microbiome Legacy Project study (23). Suspected contaminant Pseudomonas tolaasii was removed given an evident batch-associated effect. Consistency in PCR amplification bias was confirmed by assessment of the microbiome profiles from positive controls (an equimolar mixture of 20 plasmids, each containing a *cpn*60 sequence from a member of the vaginal microbiome). Assessment of identical ASVs present in both halves of all mother-infant dyads was achieved using a custom Python script (see File S2 at https://doi.org/10.6084/m9.figshare.22063148.v1) which identifies mother-infant pairs in the data set and identifies ASVs present in the maternal vaginal microbiome and one or both infant stool microbiomes at a user-defined relative abundance threshold.

### Culture screening and isolate identification.

Samples from mother-infant dyads were selected for bacterial culture if one of two criteria were met: (i) >1% of the relative abundance of a maternal vaginal microbiome was assigned to *Bifidobacterium* spp. (i.e., the maternal vaginal sample contained a sufficient abundance of bifidobacteria likely to result in successful cultivation [S. J. Dos Santos, A. C. Freitas, and J. E. Hill, unpublished observations]); (ii) identical *cpn*60 ASVs assigned to *Bifidobacterium* spp. were present in the maternal vaginal microbiome and at least one infant stool microbiome. Bifidus selective medium (BSM) was prepared by dissolving 0.12 g of a proprietary supplement (Millipore-Sigma; catalog no. 83055) into 4 mL of sterile, molecular biology-grade water and mixing the solution into 1 L of autoclaved, molten BSM agar (Millipore-Sigma; catalog no. 88517). Vaginal swab fluid (100 μL initial inoculum) and infant stool samples (one loopful resuspended in 1 mL of sterile phosphate-buffered saline [PBS]) were serially diluted in PBS, and 100 μL of 10^−2^ to 10^−4^ and 10^−4^ to 10^−6^ dilutions was inoculated onto BMS agar for vaginal and stool samples, respectively. Plates were incubated at 37°C for 72 h under anaerobic conditions using GasPak sachets (BD; catalog no. 260001), and colonies were selected for subculturing on the basis of unique colony morphology. For *Enterococcus* spp., isolates were either serendipitously recovered after culture on BSM agar or selectively isolated on m-*Enterococcus* agar (m-Ent; Millipore-Sigma; catalog no. 17185). Samples were screened for viable *Enterococcus* spp. by selective culture on m-Ent if identical *cpn*60 ASVs aligning to *Enterococcus* were identified in both maternal vaginal microbiomes and infant stool microbiomes. Freezer stocks of subcultured isolates were created by overnight culture in 2 mL of Difco clostridial broth (Millipore-Sigma; catalog no. 91365) at 37°C under anaerobic conditions, followed by mixing with 2× freezing medium (5% skim milk powder, 200 mg d-glucose, 20% glycerol) in cryovials.

A 526-bp region of the 16S rRNA gene was amplified by colony PCR from subcultured isolates using previously published primers (F1/R2 [69]) (each 50-μL reaction mixture: 1× PCR buffer, 2.5 mM MgCl_2_, 200 nM deoxynucleoside triphosphates [dNTPs], 400 nM each primer, 2 U AccuStart *Taq* [QuantaBio; catalog no. 95141]). Sterile toothpicks were used to inoculate the PCR master mix with colony material, and reaction mixtures were cycled using the following program: 95°C for 5 min, 40 cycles of 95°C for 30 s, 60°C for 30 s, and 72°C for 30 s, and 72°C for 10 min. Amplicons underwent gel purification using a QIAquick gel extraction kit (Qiagen; catalog no. 28704) and were sequenced using the amplification primers (Macrogen, Inc., Seoul, South Korea). Resultant .ab1 sequencing files were trimmed for quality and assembled using the Staden package before taxonomic identification of sequences using the Ribosomal Database Project’s SeqMatch tool (70).

### PFGE.

*Bifidobacterium* and *Enterococcus* isolates were selected for strain typing if the same species was cultured from both halves of a maternal-infant dyad. PFGE of bifidobacteria was performed on a CHEF-DR III system (Bio-Rad, Hercules, CA, USA) according to the method of Briczinski and Roberts (71) with the following modifications: 40 U of XbaI was used for digestion (2 h at 37°C), and premade 10× Tris-borate-EDTA (TBE) (Millipore-Sigma; catalog no. 11666703001) was diluted to 0.5× prior to use in electrophoresis. Also, PFGE conditions were as follows: initial switch time zero, 2 s; final switch time, 35.4 s; 6 V/cm; angle, 120° for 19.5 h with cooling at 14°C. For enterococci, standard CDC protocols for Gram-positive organisms (72) were followed with the following modifications: cell suspension buffer consisted of 10 mM Tris-HCl, 20 mM NaCl, and 50 mM EDTA, with a final pH of 7.2; lysis buffer consisted of 10 mM Tris-HCl, 50 mM NaCl, 0.2% sodium deoxycholate, 0.1% Sarkosyl, and 50 mM EDTA, with a final pH of 7.2; Tris-EDTA (TE) wash buffer consisted of 10 mM Tris-HCl and 0.1 mM EDTA, with a final pH of 7.2; 40 U of SmaI was used for digestion (2 h at 25°C); premade 10× TBE (Millipore-Sigma; catalog no. 11666703001) was diluted to 0.5× prior to use in electrophoresis. In addition, PFGE conditions were as follows: initial switch time, 3.5 s; final switch time, 23.5 s; 6 V/cm; angle, 120° for 19.5 h with cooling at 14°C. Agarose gels were stained in 400 mL of 1 μg/mL ethidium bromide for 1 h with gentle agitation and destained in an equal volume of fresh, sterile ultrapure water for 1 h before visualization on a GelDoc XRS+ system (Bio-Rad, Hercules, CA, USA).

### Whole-genome sequencing and data analysis.

A total of 35 *Bifidobacterium* isolates from 12 mother-infant dyads were selected for whole-genome sequencing based on PFGE banding patterns representing 10 pairs harboring identical strains between mothers and infants and 2 pairs with different strains. Total genomic DNA was extracted using a modified salting-out procedure (73), and DNA quality (*A*_260_/*A*_280_ ratio) was assessed using a Nanodrop spectrophotometer (Thermo Scientific, Waltham, MA, USA). Sequencing libraries were prepared using a Nextera XT DNA library prep kit according to the manufacturer’s instructions (74) and sequenced on an Illumina MiSeq using a v3 600-cycle flow cell. Sequence reads were trimmed for quality (Trimmomatic; minimum Phred score of Q20, minimum length of 50 bp), and summary statistics, including total read numbers and median read length, were extracted using SeqKit (75). Genomes were assembled from forward and reverse paired and unpaired reads using SPAdes (76) operating with the “careful” flag and k-mers of 21, 33, 55, 77, 99 and 127 bases. Quality-trimmed reads were mapped to draft assemblies using Bowtie2 (77), and coverage was calculated from indexed, sorted BAM files using SAMtools (78) (view, sort, index, and coverage). Contigs from draft assemblies were screened for contamination using Kraken2 (79) (k2_standard_20220926 prebuilt database), and any contigs not classified as *Bifidobacterium* were removed.

Complete reference genomes were downloaded in FASTA and GFF format from NCBI GenBank (B. breve ACS-071-V-Sch8b, B. longum subsp. *longum* KACC 91563, and B. bifidum PRL2010). ANI (80) was calculated pairwise across all genomes using ANIclustermap, and the resulting ANI matrix and dendrogram were plotted in RStudio (81) (R v4.1.1). Genome annotation was conducted with Prokka (82), using the above annotated reference genomes, and output .gff files served as input for pangenome analysis with Roary (83). A dendrogram was constructed using the unweighted pair group method with arithmetic mean (UPGMA) based on the presence/absence gene matrix output by Roary. A whole-genome alignment of B. breve isolate genomes produced by Snippy (84) was used to build a pairwise SNP distance matrix with snp-dists (85) and an SNP-based phylogenetic tree using FastTree (86).

### Statistical analysis.

Differences in the mean number of potential transfer events among infants delivered vaginally and by elective and emergency CS were assessed by a Kruskal-Wallis test followed by Dunn’s multiple-comparison correction. Differences in the distribution of identical and nonidentical PFGE banding patterns based on delivery mode, intrapartum antibiotic exposure, and sampling time point were assessed by chi-square test using Yates’ correction. Differences in the mean number of between- and within-dyad pairwise SNPs were calculated by the Mann-Whitney U test. All analyses were conducted in GraphPad Prism v9.3.1.

### Data availability.

Microbiome profiling data associated with this study have been deposited in the NCBI Sequence Read Archive (BioProject no. PRJNA824125). Draft genome assemblies generated in this study were deposited in GenBank and are available under BioProject no. PRJNA912610. The ASV read count table and metadata for microbiome profiles used in this study, the custom Python script used for assessing “transfer” in the microbiome data, and the input tables for the custom Python script and summary output data of ASV “transfer,” as well as summary data regarding ANI and SNP analysis from draft genome assemblies, are available to download from FigShare (see Files S1 to S4 at https://doi.org/10.6084/m9.figshare.22063148).
