# Supplementary material for: Birth Mode Does Not Determine the Presence of Shared Bacterial Strains between the Maternal Vaginal Microbiome and the Infant Stool Microbiome
Source: Microbiol Spectr. 2023 Jun 20;11(4):e00614-23. doi: 10.1128/spectrum.00614-23 (PMC10433807; doi:10.1128/spectrum.00614-23)
Supplement: Supplemental file 1 — Fig S1 to S3 and Tables S1 and S2. Download spectrum.00614-23-s0001.pdf, PDF file, 7.3 MB [file spectrum.00614-23-s0001.pdf]

Supplementary Table S1 ASVs of *Lactobacillus* spp. and *Enterococcus* spp. undergoing potential 'transfer': Taxonomic assignments of the seven *Lactobacillus* ASVs (top) and three *Enterococcus* ASVs (bottom) potentially 'transferred' to the neonatal gut. Sequence similarity (% ID) of each ASV to their corresponding closest match in cpnDB is indicated alongside the number of maternal samples above the 'transfer' threshold and the number of 'transfer' events across the dataset. 'Transfers' are broken down by infant stool sampling timepoint. ASV IDs are not consistent across tables.

ASV ID	Taxonomic assignment	% ID	Maternal samples >threshold	No. 'transfers'	Infant samples > threshold			Abundance threshold
					Both	10d only	3m only	
1	*Lactobacillus crispatus*	100	380	61	0	57	4	>0.1
2	*Lactobacillus gasseri*	100	146	12	0	7	5	
3	*Lactobacillus jensenii*	100	203	7	0	7	0	
4	*Lactobacillus paragasseri*	100	21	3	0	1	2	
5	*Lactobacillus iners*	100	97	2	0	2	0	
6	*Lactobacillus iners*	99.3	66	2	0	2	0	
7	*Lactobacillus mullieris*	100	71	2	0	2	0	

ASV ID	Taxonomic assignment	% ID	Maternal samples >threshold	No. 'transfers'	Infant samples > threshold			Abundance threshold [a]
					Both	10d only	3m only	
1	*Enterococcus casseliflavus*	100	37	3	0	0	3	>0.01
2	*Enterococcus faecalis*	100	7	3	0	2	1	
3	*Enterococcus faecalis*	99.3	6	1	0	1	0	

[a] Lower abundance threshold than for other ASV analyses due to very low *Enterococcus* abundance in vaginal microbiomes

A

B

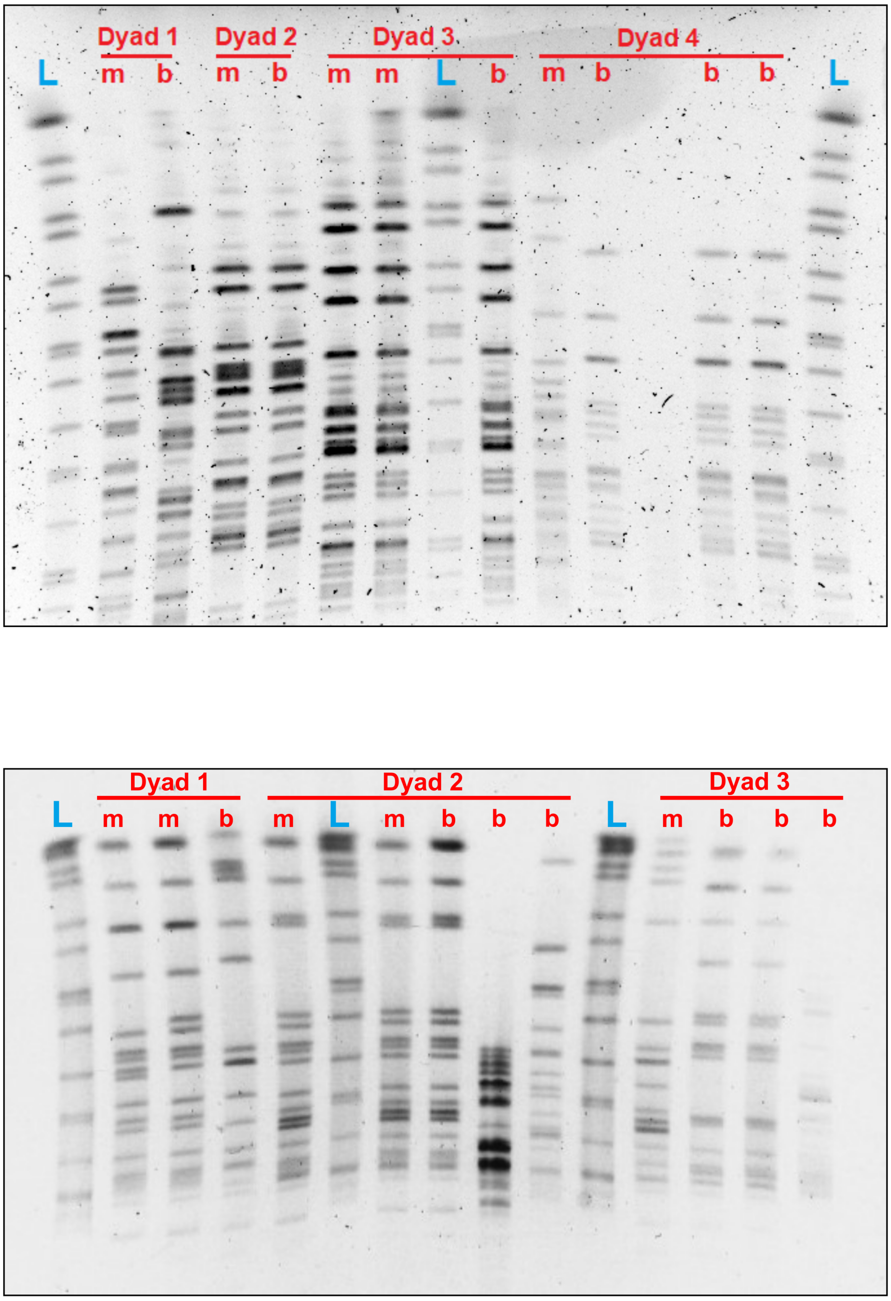

Supplementary Figure S1 Representative PFGE images for *Bifidobacterium* spp. and *Enterococcus faecalis*: Gel images representative of typical PFGE runs for isolates of (**A**) *Bifidobacterium* spp. digested with *XbaI* and (**B**) *Enterococcus faecalis* digested with *SmaI*. Isolates from the same mother-infant pairs are indicated. 'm' = maternal isolate, 'b' = baby isolate, 'L' = *Salmonella braenderup* digested with *XbaI* (ladder).

Supplementary Table S2 Summary of whole genome sequencing quality control and assembly statistics: Assembly and quality control statistics from 35 bifidobacterial draft genomes in this study. Dyad and isolate IDs are indicated, along with basic QUAST statistics, total read numbers and mean length, coverage information from samtools and number of contigs remaining after removal of non-bifidobacterial contigs

Dyad # (species)	Isolate ID	Ident. to maternal? [a]	QUAST statistics N50	L50	% GC	Length	Total no. reads [b]	Mean read length (bp) [c]	Coverage [d]	No. contigs [e]
1 (*B. breve*)	1057-dR	-	222,831	4	58.59	2,330,484	937,052	182	91.4	36
	1059-dR	Yes	222,830	4	58.59	2,330,604	791,234	179	81.9	38
	1060-OWr	Yes	222,830	4	58.59	2,329,936	1,245,875	180	123.8	35
2 (*B. bifidum*)	1424-Owp	-	364,536	3	62.63	2,186,279	821,996	178	43.2	59
	1425-Owp	Yes	364,536	3	62.64	2,182,980	686,120	177	38.7	45
3 (*B. breve*)	1439-RL	-	503,329	2	58.61	2,237,858	621,016	176	36.0	32
	1440-Owr	Yes	309,104	3	58.62	2,237,199	955,897	181	57.9	29
	1441-Owr	Yes	294,066	3	58.62	2,238,913	657,283	178	38.3	32
4 (*B. breve*)	1701-OWr1	-	569,288	2	58.74	2,363,732	950,958	179	54.2	26
	1702-OWr1	Yes	511,182	2	58.74	2,365,422	950,969	179	26.1	23
	1703-OWr1	Yes	511,084	3	58.74	2,364,907	929,249	181	48.9	28
5 (*B. breve*)	1919-OWr1	-	648,448	2	58.59	2,263,459	953,315	180	38.3	17
	1920-OWr1	Yes	1,936,625	1	58.59	2,274,310	868,341	179	54.8	30
	1921-OWr1	Yes	1,936,799	1	58.60	2,280,291	776,189	177	36.1	42
6 (*B. longum*)	1927-OWp1	-	213,260	4	59.94	2,310,479	871,063	179	78.2	61
	1928-OWp1	No	97,629	9	60.11	2,362,531	959,578	179	107.0	82
	1929-OWp1	No	94,984	10	60.11	2,363,795	904,407	178	94.7	87
7 (*B. breve*)	509-OW	-	324,392	3	58.70	2,373,647	993,925	180	89.3	35
	511-OW	Yes	275,883	3	58.70	2,376,484	980,898	182	63.6	48
	512-OW	Yes	275,892	3	58.70	2,373,451	782,740	179	86.9	28
	512-W	No	648,613	2	58.58	2,266,399	951,922	182	51.5	28
8 (*B. breve*)	734-OW	-	222,830	4	58.72	2,362,298	1,101,416	180	106.0	33
	736-OW	Yes	221,036	5	58.74	2,372,380	779,484	176	73.0	54
9 (*B. breve*)	744-OW	-	585,010	2	58.80	2,293,730	1,162,188	182	105.0	17
	744-W	-	584,996	2	58.81	2,293,631	1,297,248	183	94.9	19
	746-OW	No	318,463	3	58.83	2,342,067	980,960	182	79.6	42
	746-W	No	318,462	3	58.82	2,340,838	940,551	180	75.5	43
10 (*B. breve*)	832-OWp	-	613,380	2	58.91	2,238,393	833,804	178	53.2	10
	834-OWp	Yes	1,562,671	1	58.91	2,239,343	1,082,755	180	78.2	15
	835-OWp	Yes	1,357,653	1	58.90	2,238,744	948,508	180	81.9	13
11 (*B. breve*)	962-Irr	-	1,698,826	1	58.64	2,354,534	910,935	179	31.1	9
	964-W-Irr	Yes	1,699,034	1	58.64	2,353,524	1,035,409	179	48.1	6
12 (*B. breve*)	965-OWr	-	366,304	3	58.67	2,361,091	1,016,991	179	73.9	36
	967-OWr	Yes	366,305	3	58.68	2,360,819	1,067,271	180	69.4	41
	968-OWr	Yes	366,302	3	58.67	2,360,492	1,279,268	182	98.2	43
	**Mean**		**595,126**	**3**	**59.04**	**2,312,144**	**943,623**	**179**	**69**	**35**

[a] Identical banding patterns shared between infant isolate and at least one maternal isolate

[b] Sum of paired and unpaired forward and reverse reads used for assembly with 'spades'

[c] Mean calculated from median read lengths from paired and unpaired forward and reverse reads output by 'seqkit stats'

[d] Mean coverage per contig calculated by mapping reads onto assembled contigs using 'bowtie2' and 'samtools coverage'

[e] Number of contigs remaining after removal of non-bifidobacterial contigs by 'kraken2'

A

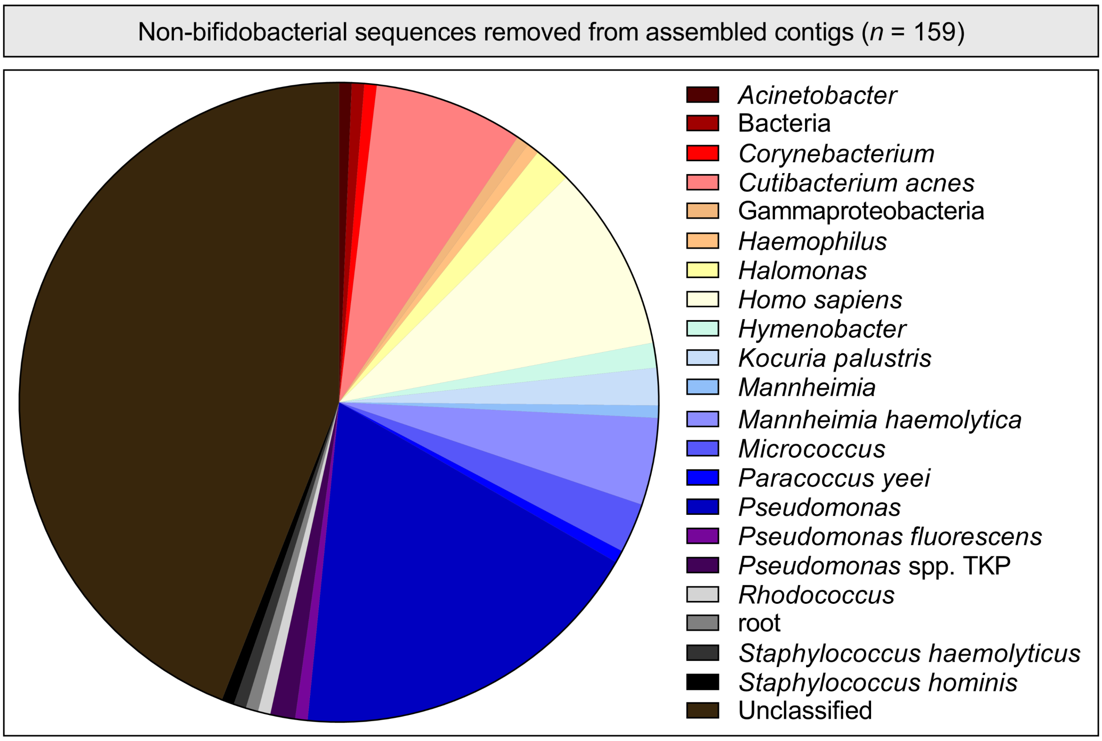

B

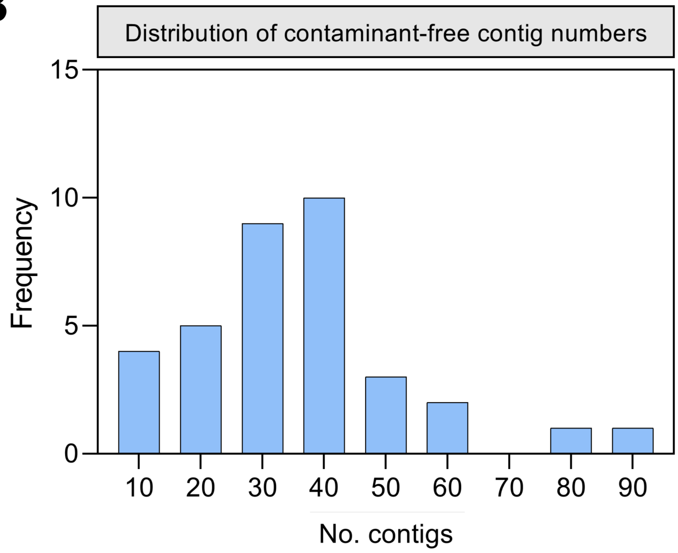

Supplementary Figure S2 Contamination removed from draft-level assemblies of 35 bifidobacterial genomes: Genome assemblies were screened for contaminant contigs using Kraken2 and non-bifidobacterial sequences were removed. (**A**) Classification of contigs aligning to non-bifidobacterial taxa. (**B**) Distribution of the number of contigs per genome across 35 draft-level assemblies.

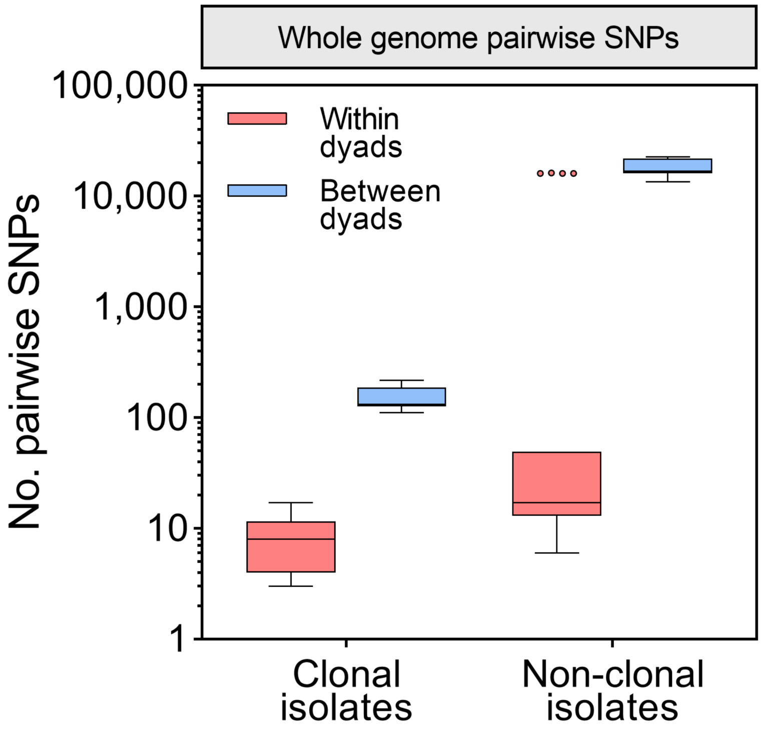

Supplementary Figure S3 Numbers of within-dyad SNPs are consistently lower than between-dyad SNPs even among highly-related *B. breve* isolates: The number of SNPs between isolates for all comparisons within (red) and between (blue) mother-infant pairs. Isolates were separated into two sub-populations based on pairwise SNPs: 'clonal' isolates (top-left quadrant, Fig. 4A) and non-clonal isolates (bottom-right quadrant, Fig. 4A). Bar represents median number of SNPs, whiskers indicate interquartile range.
